# The highly conserved 5' untranslated region as an effective target towards the inhibition of Enterovirus 71 replication by unmodified and appropriate 2'-modified siRNAs

**DOI:** 10.1186/1423-0127-19-73

**Published:** 2012-08-13

**Authors:** Jun-Xia Deng, Xiao-Jing Nie, Ying-Feng Lei, Chao-Feng Ma, Dong-Liang Xu, Biao Li, Zhi-Kai Xu, Guo-Cheng Zhang

**Affiliations:** 1 Department of Pediatrics, Xijing Hospital, Fourth Military Medical University, 15th Changlexi Road, Xi′an, 710032, P. R. China; 2 Department of Microbiology, Fourth Military Medical University, 17th Changlexi Road, Xi′an, 710032, P. R. China; 3 Department of Viral Diseases Laboratory, Xi′an Center for Disease Control and Prevention, 599th Xiying Road, Xi′an, 710054, P. R. China

**Keywords:** Enterovirus 71, RNA interference, siRNA, 5^′^ UTR, Viral inhibition, Antiviral agent, 2^′^-O-methylation modification, 2^′^-fluoro modification

## Abstract

**Background:**

Enterovirus 71 (EV71) is a highly infectious agent that plays an etiological role in hand, foot, and mouth disease. It is associated with severe neurological complications and has caused significant mortalities in recent large-scale outbreaks. Currently, no effective vaccine or specific clinical therapy is available against EV71.

**Methods:**

Unmodified 21 nucleotide small interfering RNAs (siRNAs) and classic 2^′^-modified (2^′^-O-methylation or 2^′^-fluoro modification) siRNAs were designed to target highly conserved 5^′^ untranslated region (UTR) of the EV71 genome and employed as anti-EV71 agents. Real-time TaqMan RT-PCR, western blot analysis and plaque assays were carried out to evaluate specific viral inhibition by the siRNAs.

**Results:**

Transfection of rhabdomyosarcoma (RD) cells with siRNAs targeting the EV71 genomic 5^′^ UTR significantly delayed and alleviated the cytopathic effects of EV71 infection, increased cell viability in EV71-infected RD cells. The inhibitory effect on EV71 replication was sequence-specific and dosage-dependent, with significant corresponding decreases in viral RNA, VP1 protein and viral titer. Appropriate 2^′^-modified siRNAs exhibited similar RNA interference (RNAi) activity with dramatically increased serum stability in comparison with unmodified counterparts.

**Conclusion:**

Sequences were identified within the highly conserved 5^′^ UTR that can be targeted to effectively inhibit EV71 replication through RNAi strategies. Appropriate 2^′^-modified siRNAs provide a promising approach to optimizing siRNAs to overcome barriers on RNAi-based antiviral therapies for broader administration.

## Background

Enterovirus 71 (EV71) has been implicated in numerous epidemics of hand, foot, and mouth disease (HFMD), which primarily affects infants and young children, resulting in the appearance of vesicular rashes on hands, feet, and oral mucosa. More importantly, EV71-infected children can develop neurological complications, such as aseptic meningitis, acute flaccid paralysis, encephalitis, acute cerebellar ataxia, brainstem encephalitis, as well as fatal pulmonary edema or cardiopulmonary collapse [1]. Currently, EV71 is recognized as the most clinically important neurotropic virus since poliovirus has been nearly eradicated in the majority of countries around the world [2]. Significant morbidities and mortalities associated with increasingly frequent HFMD outbreaks throughout the Asia-Pacific region have raised public concerns [3-6]. Despite improvements in virological and medical research, treatments for acute EV71 infections are mainly aimed towards alleviating clinical symptoms, and there is still no specific antiviral agent available against EV71.

RNA interference (RNAi) is a biological and specific post-transcriptional gene silencing mechanism that has been employed in the development of numerous promising therapeutic agents [7]. Since the initial report of RNAi-mediated inhibition of the human respiratory syncytial virus in 2001 [8], a variety of other viruses have been successfully targeted using RNAi strategies [9-13]. Several RNAi-based antiviral therapeutics are currently in various phases of clinical trials for the treatment of viral infections, such as the human immunodeficiency virus, respiratory syncytial virus, and hepatitis C virus [14]. As a positive-stranded RNA virus, EV71 is an attractive target for RNAi therapy due to the functions of its genome as both a replication template and messenger RNA [15]. The destruction of EV71 RNA could eliminate not only protein synthesis but also viral replication.

In spite of the immense attractiveness of efficient gene knockdown as a therapeutic strategy, RNAi applications still face several critical hurdles that have not yet been comprehensively addressed. Identifying the appropriate chemical modifications will be essential to resolving these hurdles and bringing RNAi to the clinic [16]. The properties of small interfering RNAs (siRNAs) can be optimized by incorporation of chemically modified RNA analogues to increase nuclease resistance, improve target specificity and maintain gene-silencing activity [16]. Importantly, the high efficacy of chemically modified siRNAs can be accompanied by low cell toxicity [17]. Recent studies have successfully improved siRNA performance by incorporation of chemically modified RNA analogues against virus infection [18-22]. Among these, 2’-O-methylation (2’-OMe) and 2’-fluoro (2’-F) are classic patterns of chemical modification.

The selection of appropriate siRNA targets is a critical initial step in the development of antiviral RNAi therapeutics. Successful siRNA designs must be based on the conserved sequences of the viral genome. EV71 is a small, non-enveloped, positive single-stranded RNA virus belonging to the genus *Enterovirus* within the family *Picornaviridae*. Like all picornavirus, EV71 genome possesses a conserved 5^′^ untranslated region (UTR) of approximately 740 nucleotides (nt) [23,24]. The 5^′^ UTR of EV71 is involved in key steps of the viral life cycle, including replication and translation [25,26]. Due to its high degree of conservation which renders it less likely to mutate and functional importance for viral replication, the 5^′^ UTR of the EV71 genome is an attractive target region for siRNA therapeutic treatment. In both cell cultures and animal experiments, RNAi in the form of chemically synthesized siRNA or plasmid-derived shRNA has been used to effectively inhibit EV71 infection, revealing several potential target sequences within the 3^′^ UTR, VP1, 3D, 3C, and 2C regions of the EV71 genome [27-31]. However, the higher order structure of the EV71 genomic 5^′^ UTR makes targeting of this region especially challenging. To date, no effective siRNAs targeting the conserved 5^′^ UTR of the EV71 genome have been reported.

The application of unmodified 21 nt siRNAs and 2^′^-modified (2^′^-OMe and 2^′^-F) siRNAs to target highly conserved 5^′^ UTR of the EV71 genome for specific viral inhibition was investigated in this study. Identification of effective target sequences within the highly conserved 5^′^ UTR and optimization of siRNA properties by 2^′^-OMe or 2^′^-F modification for future *in vivo* applications were considered primary objectives. Direct *in vitro* evaluation explored the effectiveness of siRNAs targeting the 5^′^ UTR of the EV71 genome in inhibiting viral replication. Meanwhile, we observed good compatibility of the introduction of appropriate 2^′^-OMe or 2^′^-F modifications into the siRNA duplexes, which maintained high antiviral activities. Enhanced serum stability was confirmed as a major advantage of the 2^′^-modified siRNAs in comparison with unmodified counterparts. These strategies may ultimately lead to the development of appropriate chemically modified siRNAs against clinical EV71 infection.

## Methods

### Cell culture and virus strain

Rhabdomyosarcoma (RD) cells were routinely grown in Dulbecco’s modified Eagle’s medium (DMEM) supplemented with 10% fetal bovine serum (FBS, Gibco BRL, Grand Island, NY, USA). The EV71 strain (Genbank accession no. HM003207.1) was provided by the Department of Viral Diseases Laboratory, Xi^′^an Center for Disease Control and Prevention (Xi^′^an Shanxi, People^′^s Republic of China). Viruses were propagated and titrated in RD cells.

### Design of unmodified and 2^′^-modified 21 nt double-stranded siRNAs

Initially, 21 nt double-stranded siRNAs containing 19-mer core sequences and 2d-TT at the 3^′^ ends were designed to target the 5^′^ UTR of the EV71 genome. The siRNA screening procedure was primarily based on a combination of RNA target accessibility prediction, siRNA duplex thermodynamic properties, and empirical design rules [32] using the web-based tools (http://sfold.wadsworth.org/cgi-bin/sirna.pl and http://www.genebee.msu.su/services/rna2_reduced.html). The nucleotide identities between siRNA targeting sequences in HM003207.1 and corresponding sequences in other EV71 China strains available in GenBank were also considered by using the cluster alignment function of the DNAStar (DNAStar, Inc., Madison, WI, USA) software. Specifically identified sequences were subjected to analysis by the BLAST algorithm (http://www.ncbi.nlm.nih.gov/BLAST) to exclude homology in both human and mouse genomes. Unmodified siRNAs targeting the 115–133 nt sequence and the 648–666 nt sequence of 5^′^ UTR that satisfied the above criteria were designated as si-1 and si-2. Subsequently, si-1 and si-2 were chemically modified by 2^′^-OMe or 2^′^-F at U and C sequences on complementary strands. The 2^′^-modified siRNAs were designated as si-1OMe, si-1 F, si-2OMe, and si-2 F. A scrambled sequence with the same base composition as si-2 and no sequence homology to the viral genome was designed as a negative control. In addition, FAM-labeled si-2 was synthesized with the label on the 5^′^ end of the sense strand, designated as si-2FAM. The siRNA sequences are listed in Table 1. All siRNAs were synthesized by Genepharma Co. Ltd., Shanghai, China.

**Table 1:** Nucleotide sequences of siRNAs and their target positions in the EV71 genome (Genbank accession no. HM003207.1)

**siRNA**	**Chemically modified**	**Nucleotide sequence**	**Genomic position**
si-1	No	5^′^-CAGCAAACCACGAUCAAUATT-3^′^(Sense)	115-133
		5^′^-UAUUGAUCGUGGUUUGCUGTT-3^′^(Anti-sense)	
si-1OMe	2^′^-OMe-modified	5^′^-*C*AG*C*AAA*CC*A*C*GA*UC*AA*U*ATT-3^′^(Sense)	115-133
		5^′^-*U*A*UU*GA*UC*G*U*GG*UUU*G*CU*GTT-3^′^(Anti-sense)	
si-1 F	2^′^-F-modified	5^′^-*C*AG*C*AAA*CC*A*C*GA*UC*AA*U*ATT-3^′^(Sense)	115-133
		5^′^-*U*A*UU*GA*UC*G*U*GG*UUU*G*CU*GTT-3^′^(Anti-sense)	
si-2	No	5^′^-CAGAGCAAUUGUUUACCUATT-3^′^(Sense)	648-666
		5^′^-UAGGUAAACAAUUGCUCUGTT-3^′^(Anti-sense)	
si -2OMe	2^′^-OMe-modified	5^′^-*C*AGAG*C*AA*UU*G*UUU*A*CCU*ATT-3^′^(Sense)	648-666
		5^′^-*U*AGG*U*AAA*C*AA*UU*G*CUCU*GTT-3^′^(Anti-sense)	
si-2 F	2^′^-F-modified	5^′^-*C*AGAG*C*AA*UU*G*UUU*A*CCU*ATT-3^′^(Sense)	648-666
		5^′^-*U*AGG*U*AAA*C*AA*UU*G*CUCU*GTT-3^′^(Anti-sense)	
scr	No	5^′^-GGAUUGACCUCUCAUAUAATT-3^′^(Sense)	
		5^′^-UUAUAUGAGAGGUCAAUCCTT-3^′^(Anti-sense)	
si-2FAM	FAM-modified	5^′^-(FAM) CAGAGCAAUUGUUUACCUATT-3^′^(Sense)	648-666
		5^′^-UAGGUAAACAAUUGCUCUGTT-3^′^(Anti-sense)	

*Italicized letters indicate the positions of chemically modified nucleotides.

### Transfection and infection

Transfection was carried out under optimal conditions. RD cells were seeded in 24-well plates (8 × 10^4^ cells/well) without antibiotics the day before transfection to allow adherence and to reach 70–80% confluence at the time of transfection. The culture medium was changed to 500 μl per well of Opti-MEM® (Gibco BRL, Grand Island, NY, USA) immediately prior to transfection. For each transfection, the appropriate amount of siRNA was diluted in 50 μl of Opti-MEM®, while 1 μl of Lipofectamine^TM^ 2000 (Invitrogen, Carlsbad, CA, USA) was also diluted in 50 μl of Opti-MEM® in another tube simultaneously. After incubation at room temperature for 5 min, these two solutions were mixed gently and incubated for 20 min at room temperature to allow the siRNA:Lipofectamine^TM^ 2000 complexes to form. Subsequently, 100 μl of each complex was added to each well and mixed by gently rocking the plate. The final concentration of siRNAs was 25 nM, 50 nM, or 100 nM. After 5 h, the medium was removed, and transfected RD cells were infected with EV71 at a multiplicity of infection (MOI) of 0.01 for 1 h. The cells were washed twice to remove any unabsorbed virus, and 500 μl of fresh maintenance medium was added to each well. Cells were then incubated at 37°C, and the morphological changes of RD cells were observed at various time points. Mock transfections were carried out as described above; however, no siRNA was added as a negative control. Cell supernatants and cell lysates were collected at designated post-infection time points and stored at −80°C for further studies.

### Fluorescence and flow cytometric analysis

Fluorescein labeled si-2FAM was used to confirm the localization of siRNA in RD cells and the optimal transfection efficiency. RD cells were seeded onto coverslips and transfected with varying concentrations (25, 50, and 100 nM) of si-2FAM. As a control, one of the wells was treated with 100 nM of si-2FAM without Lipofectamine^TM^ 2000. The medium was changed at 5 h post-transfection. At 6 h post-transfection, cells were washed twice with phosphate buffered saline (PBS) and fixed with 4% paraformaldehyde for 30 min. Cells were then washed again with PBS, and Hoechst 33258 (0.5 ug/ml; Sigma, St. Louis, MO, USA) was used to stain cell nuclei. The cells were observed using the FLUOVIEW FV1000 confocal laser scanning biological microscope (Olympus, Tokyo, Japan) equipped with the FV10-ASW system (Olympus, Tokyo, Japan).

Flow cytometry was used to precisely measure the transfection efficiency. RD cells were seeded in 6-well plates and transfected with varying concentrations (25, 50, and 100 nM) of si-2FAM. The medium was changed at 5 h post-transfection. At 6 h post-transfection, RD cells were trypsinized and resuspended in PBS. Transfection efficiency was determined by detection of fluorescein-labeled RD cells using FACSAria flow cytometry (BD Biosciences, San Jose, CA, USA).

### Cytotoxicity assay

RD cells were seeded in 96-well plates (1.6 × 10^4^ cells/well) and transfected with 100 nM of various siRNAs. At 24, 48, and 72 h post-transfection, a 20 μl volume of MTT (3-[4,5-dimethylthiazol-2-yl]-2,5-diphenyltetrazolium bromide) (5 mg/ml; Sigma, St. Louis, MO, USA) reagent was added to each cell. After incubation at 37°C for 4 h, the cell supernatant of each well was removed. A 150 μl volume of DMSO (Sigma, St. Louis, MO, USA) was added to each well, followed by 10 min of shaking. Absorbance values were measured at a test wavelength of 570 nm with a reference wavelength of 690 nm.

### Cell viability assay

RD cells were seeded in 96-well plates (1.6 × 10^4^ cells/well) and transfected with 50 nM or 100 nM of various siRNAs. At the end of the transfection period (5 h), cells were infected with 0.01 MOI of EV71 for 1 h. At 24, 36, 48, 60, and 72 h post-infection, MTT assays were carried out as described above to assess the viability of EV71-infected RD cells transfected with unmodified and 2^′^-modified siRNAs.

### Real-time TaqMan RT-PCR

Viral RNA extraction was performed as previously reported [13]. Briefly, at 36 h post-infection, RD cells were washed with PBS, and 150 μl of CelLytic^TM^ M Cell Lysis Reagent (Sigma, St. Louis, MO, USA) was added into the individual wells containing the RD cells. Viral RNA extraction was then carried out using the QIAamp® Viral RNA Mini Kit according to the manufacturer’s instructions (Qiagen, Valencia, CA, USA). Finally, RNA was eluted in 60 μl of elution buffer. Efficiencies of various siRNAs in inhibition of EV71 replication were analyzed using the real-time TaqMan RT-PCR assay. The Human Enterovirus Type 71 Fluorescence Quantitative Polymerase Chain Reaction Diagnostic Kit (Suoao Biomedtech Co. Ltd., Beijing, China) was used in this study. The kit contains a TaqMan probe (5'-FAM-TGCAAGGATGCTAGTGATATCCTGC-TAMRA-3') and a pair of primers (forward, 5'-GCAGCCCAAAAGAACTTCACT-3'; reverse, 5'-ATCTGCCACCCTATCTCCCT-3') that target the 2366–2455 nt region of the EV71 genome (Genbank accession no. HM003207.1). According to the manufacturer’s instructions, a reverse transcription reaction was carried out in a 10 μl reaction mixture containing 2 μl of total RNA, 7 μl of EV71-RT MIX, and 1 μl of reverse transcriptase at 42°C for 30 min. Subsequently, the reverse transcriptase was inactivated at 98°C for 5 min. Following reverse transcription, PCR amplification was performed in a 25 μl reaction mixture containing 3 μl cDNA, 20 μl EV71-PCR MIX, and 2 μl Taq Polymerase. The real-time TaqMan RT-PCR conditions were as follows: 1 cycle at 94°C for 2 min, 40 cycles at 93°C for 30 s, and 55°C for 45 s. EV71 RNA levels were detected using the Mx3000P real-time PCR system (StrataGene, La Jolla, CA, USA). A series of 10-fold dilutions of the standard plasmid contained in the test kit were used to obtain the standard curve used in absolute quantitation of viral RNA.

### SDS-PAGE and western blot analysis

In order to obtain sufficient viral protein, RD cells in 6-well plates infected with 0.01 MOI of EV71 were lysed with 150 μl of CelLytic^TM^ M Cell Lysis Reagent at 36 h post-infection. The protein concentration was determined by BCA reagents (Pierce, Rockford, IL, USA) according to the instructions provided by the manufacturer. A 40 μg aliquot of each cell lysate was electrophoresed in a 12% polyacrylamide gel and transferred to a PVDF membrane (Millipore, Billerica, MA, USA). Western blot was performed according to a standard protocol. Protein detection was performed using an anti-EV71 VP1 mouse polyclonal antibody (Abnova, Taipei, Taiwan) and an anti-β-actin antibody (Sigma, St. Louis, MO, USA). Anti-mouse and anti-rabbit secondary antibodies labeled with IRDye infrared dyes (LI-COR Biosciences, Lincoln, NE, USA) were used in the infrared fluorescence detection procedure. The fluorescent signals on the PVDF membrane were visualized using the Odyssey Infrared Imaging System (LI-COR Biosciences, Lincoln, NE, USA). Quantification of bands was performed using Image J software (NIH, Bethesda, MD, USA).

### Virus titering plaque assay

To ascertain the effect of siRNA-mediated intracellular inhibition of EV71 replication on the progeny virus generation, plaque assays were carried out to determine virus titers of the culture supernatants. Culture supernatants from each well were collected at 48 h post-infection and centrifuged at 1000 × g for 10 min to remove cellular debris. Confluent monolayer of RD cells in 6-well plates was inoculated with 500 μl of supernatant from each test group at a 10^-4^ dilution for 1 h. The media was aspirated off, and each well was covered with 3 ml of 1% (w/v) carboxymethylcellulose (CMC) in maintenance medium. After incubation at 37°C for 72 h, the plaque media was removed, and RD cells were fixed with 20% formalin in PBS. After fixation, the plaques were stained with 1% crystal violet stain for half an hour at room temperature, and the plaques were counted.

### Serum stability

Unmodified si-2 and corresponding 2^′^-modified si-2OMe and si-2 F were used to examine the ability of ribose chemical modifications to improve the serum stability of siRNAs. Various siRNAs were dissolved in RNase-free water containing 10% FBS, 100% mouse serum (Invitrogen, Camarillo, CA, USA), or 100% human serum (Sigma, St Louis, MO, USA) at a final concentration of 10 μM and incubated at 37°C. Aliquots of 5 μl were withdrawn at different time points (0, 0.5, 1, 3, 6, 24, 48, and 72 h) and immediately frozen in 15 μl TBE-loading buffer [TBE (0.1 M Tris–HCl pH 8.4, 2 mM EDTA, 0.09 M boric acid), 7 M urea, 0.01% xylene cyanol, 0.1% bromphenol blue]. RNAs were separated using 15% polyacrylamide-TBE under non-denaturing conditions and visualized by staining with GelRed^TM^ Nucleic Acid Gel Stain (Biotium, Hayward, CA, USA). Gels were imaged using a 300 nm transilluminator and photographed with an ethidium bromide filter and Polaroid 667 black-and-white print film (Alpha Innotech, Santa Clara, CA, USA).

### Statistical analyses

All data were expressed as means ± standard deviation (SD). Statistical analyses were performed using one-way ANOVA followed by Dunnett’s post-hoc test. Differences with a *P*-value less than 0.05 were considered to be statistically significant (*P* < 0.05).

## Results

### High transfection efficiency of siRNAs using Lipofectamine^TM^ 2000

**Figure 1:**
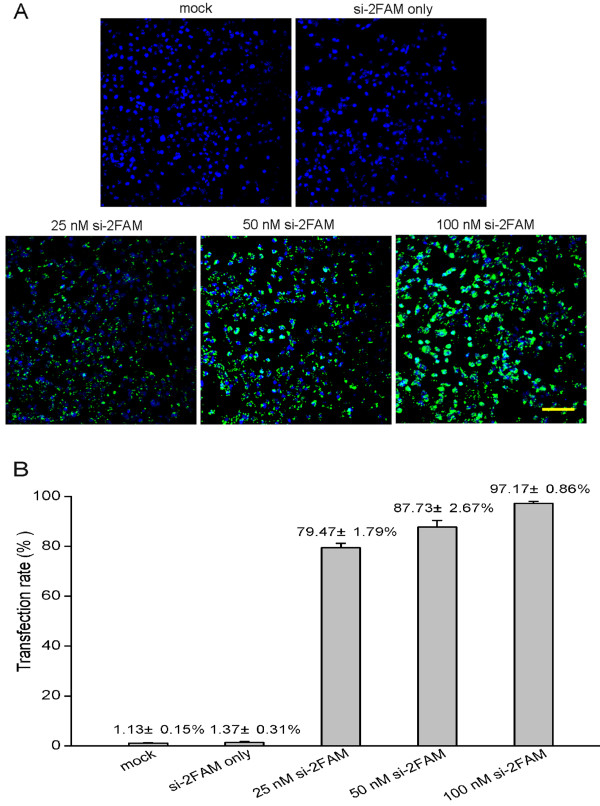
**RD cells transfected with increasing concentrations of fluorescein labeled si-2FAM.** (**A**) Representative confocal microscopy images of RD cells transfected with 25, 50, or 100 nM of si-2FAM are shown, as indicated. Cell nuclei were stained with Hoechst 33258 dye. The image marked ‘si-2FAM only’ indicates RD cells treated with 100 nM si-2FAM without Lipofectamine^TM^ 2000. The mock transfection control was carried out without the addition of si-2FAM. Bar = 100 μm. (**B**) Transfection efficiency of different concentration of si-2FAM. The proportions of fluorescein-labeled RD cells transfected with 25, 50, or 100 nM si-2FAM at 6 h post-transfection were shown using FACSAria flow cytometry. All data are presented as means ± SD from three independent experiments.

At 6 h post-transfection, fluorescein labeled si-2FAM could be seen localized in the cytoplasm of RD cells (Figure 1A). With increasing si-2FAM concentrations, a corresponding increase of si-2FAM amounts in RD cells was observed (Figure 1A). Conversely, no fluorescence was observed in the RD cells when 100 nM si-2FAM was transfected in the absence of Lipofectamine^TM^ 2000 (Figure 1A). These observations indicated that the fluorescence was not due to random adherence of siRNAs to the membrane of RD cells, verifying the successful transfection of siRNAs into RD cells by Lipofectamine^TM^ 2000. Furthermore, flow cytometry provided precise measurements of transfection efficiency by enumerating fluorescein-labeled RD cells. At 6 h post-transfection, the percentages of fluorescein-labeled cells treated with si-2FAM at 25 nM, 50 nM, and 100 nM were 79.47 ± 1.79%, 87.73 ± 2.67%, and 97.17 ± 0.86%, respectively (Figure 1B). Therefore, siRNA concentrations at 50 nM and 100 nM were determined to be sufficient and used for the following RNAi experiment.

### Unmodified and 2'-modified siRNAs showed no toxicity in RD cells

**Figure 2:**
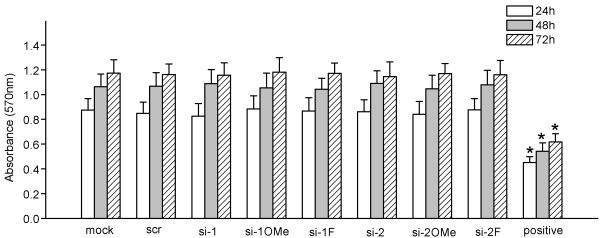
**Cytotoxicity assay of RD cells transfected with unmodified and 2′-modified siRNAs.** RD cells were transfected with 100 nM of various siRNAs. The mock transfection control was carried out without the addition of siRNA. ‘Scr’ represents RD cells transfected with 100 nM of scrambled siRNA using 0.25 μl of Lipofectamine^TM^ 2000. Positive control represents RD cells transfected with 2.5 μl of Lipofectamine^TM^ 2000. All data are presented as means ± SD from three independent experiments, each performed in triplicate. ^*^P < 0.05, compared with mock transfection control at 24, 48, and 72 h post-transfection, respectively. The data from the cells transfected with unmodified and 2′-modified siRNAs showed no significant difference compared to the mock transfection control (*P* > 0.05).

The potential cytotoxicity of unmodified and 2^′^-modified siRNAs was detected by MTT assays carried out at 24, 48, and 72 h post-transfection. The results showed that all the siRNAs used in this study, regardless of the observed time point or chemical modification, did not exhibit any cytotoxic effects which could affect the growth and viability of RD cells, even at the concentration of 100 nM (Figure 2).

### Unmodified and appropriate 2^′^-modified siRNAs delayed cytopathic effects (CPE) and increased cell viability in EV71-infected RD cells

**Figure 3:**
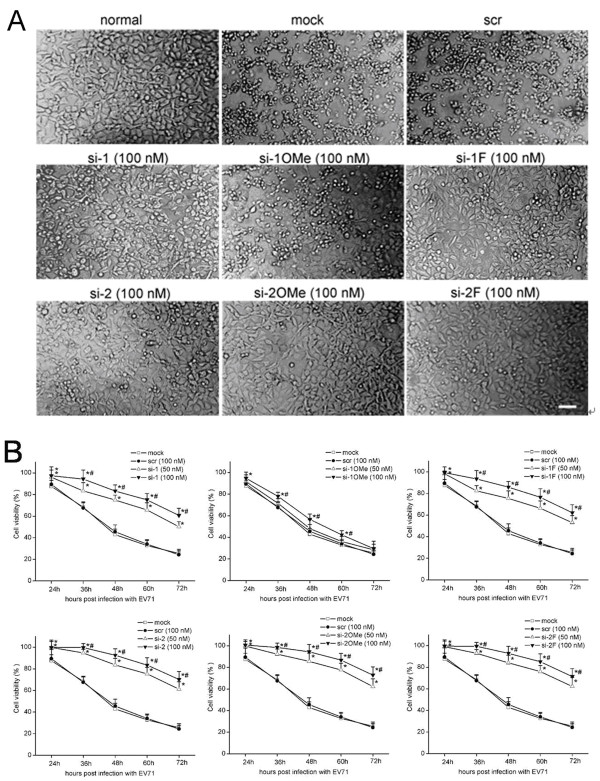
**Protection of RD cells from EV71-induced CPE and increased cell viability.** RD cells were transfected with unmodified and 2′-modified siRNAs targeting the 5′ UTR of the EV71 genome and then infected with 0.01 MOI of EV71. (**A**) Morphological changes in RD cells transfected with 100 nM various siRNAs at 48 h post-infection are shown. ‘Normal’ represents non-infected normal cells. Cells treated only with Lipofectamine^TM^ 2000 (mock transfection) and cells transfected with 100 nM of scrambled siRNA (scr) and then infected with EV71 served as negative controls. Three independent experiments were performed. Bar = 100 μm. (**B**) The viability of RD cells at 24, 36, 48, 60, and 72 h post-infection was evaluated by MTT assays. Viability percentage values shown are means ± SD from three independent experiments, each performed in triplicate. ^*^P < 0.05, compared with mock transfected control. ^#^*P* < 0.05, compared with 50 nM of si-1, si-1OMe, si-1 F, si-2, si-2OMe, and si-2 F, respectively.

RD cells were transfected with unmodified and 2^′^-modified siRNAs to evaluate their protective effect through observation of CPE in EV71-infected RD cells. Morphological changes in RD cells were observed with phase-contrast microscopy at different time points. At 24 h post-infection, the mock transfection cells and scrambled siRNA transfected cells exhibited CPE, characterized by increased shrinkage and refraction, in contrast with non-infected normal cells. Meanwhile, no CPE was observed by microscopic examination in EV71-infected cells transfected with either unmodified (si-1, si-2) or 2^′^-modified (si-1 F, si-2OMe, si-2 F) siRNAs; however, cells transfected with si-1OMe showed slight CPE. At 36 h post-infection, cells transfected with 50 nM of si-1 or si-1 F showed slight CPE. At 48 h post-infection, cells transfected with si-2, si-2OMe, or si-2 F showed slight CPE, whereas mock transfection cells, scrambled siRNA transfected cells, and si-1OMe transfected cells showed complete CPE, characterized by shrinkage, enhanced refraction, rounding up, and detachment from the culture plate (Figure 3A). The siRNAs (si-2, si-2OMe, and si-2 F) targeting the 648–666 nt sequence of the EV71 genomic 5^′^ UTR conferred a remarkable level of resistance to infection, thus delaying CPE in EV71-infected RD cells up to 48 h post-infection. These inhibitory effects lasted up to 72 h post-infection. The viral inhibitory effect of siRNAs (si-1 and si-1 F) targeting the 115–133 nt sequence of the EV71 genomic 5^′^ UTR was also evident in RD cells, delaying CPE for up to 36 h post-infection. The scrambled siRNA failed to protect against CPE in EV71-infected RD cells even at the concentrations of 100 nM (Figure 3A).

The protective effect of the unmodified and 2^′^-modified siRNAs over a period of time after EV71 infection was also evaluated by cell viability assay. At 24, 36, 48, 60, and 72 h post-infection, viability percentages indicated that an obvious elevation of viability could be achieved in RD cells transfected with unmodified (si-1, si-2) or 2^′^-modified (si-1 F, si-2OMe, si-2 F) siRNAs compared to mock transfection cells and scrambled siRNA transfected cells (Figure 3B). However, 2^′^-modified si-1OMe was not as effective as unmodified si-1, as there was only a slight elevation of cell viability in EV71-infected RD cells transfected with si-1OMe (Figure 3B). RD cells transfected with effective siRNAs at the concentration of 100 nM were found to exhibit higher viabilities compared with those observed upon treatment with the concentration of 50 nM (Figure 3B). Prolonged incubation up to 72 h post-infection was shown to lead to increased cell death (Figure 3B).

### Reduction of EV71 RNA transcripts by unmodified and appropriate 2^′^-modified siRNAs

**Figure 4:**
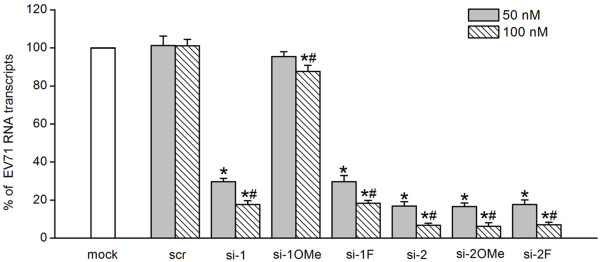
**Real-time TaqMan RT-PCR analyses of EV71 RNA transcripts.** RD cells were transfected with unmodified and 2′-modified siRNAs targeting the 5′ UTR of the EV71 genome and then infected with 0.01 MOI of EV71. At 36 h post-infection, viral RNA was extracted from the RD cells. The EV71 RNA transcript levels are shown relative to the mock transfection control in percentages. All data are presented as means ± SD from three independent experiments, each performed in duplicate. ^*^P < 0.05, compared with mock transfection control. ^#^*P* < 0.05, compared with 50 nM of si-1, si-1OMe, si-1 F, si-2, si-2OMe, and si-2 F, respectively.

EV71 RNA transcript levels after treatment with unmodified and 2^′^-modified siRNAs before EV71 infection were analyzed by real-time TaqMan RT-PCR assay. Treatment of RD cells with unmodified (si-1, si-2) and 2^′^-modified (si-1 F, si-2OMe, or si-2 F) siRNAs targeting the 5^′^ UTR of the EV71 genome resulted in a dosage-dependent decrease in EV71 RNA transcripts compared with the mock transfection control (Figure 4). The siRNAs (si-2, si-2OMe, and si-2 F) targeting the 648–666 nt sequence of the EV71 genomic 5^′^ UTR achieved more effective inhibition of replication as evidenced by the greater reduction in copies of EV71 RNA transcripts, compared with the siRNAs (si-1 and si-1 F) targeting the 115–133 nt sequence of the EV71 genomic 5^′^ UTR at the same concentrations (Figure 4). EV71 RNA transcript levels were reduced to 16.84 ± 2.30% and 6.80 ± 1.04% following the transfection of RD cells with 50 nM and 100 nM of si-2, respectively, compared with that in the mock transfection control cells (Figure 4). The 2^′^-modified si-2OMe and si-2 F were also fairly efficient in reducing EV71 RNA transcripts. EV71 RNA transcript levels declined to 16.56 ± 1.99% and 6.22 ± 1.85% with 50 nM and 100 nM of si-2OMe, respectively, and 17.63 ± 2.45% and 7.02 ± 1.38% with 50 nM and 100 nM of si-2 F, respectively (Figure 4). When the RD cells were treated with si-1 and si-1 F, the levels of EV71 RNA transcripts were found to be, respectively, 29.72 ± 1.72%, 29.72 ± 3.16% (50 nM) and 17.64 ± 2.03%, 18.34 ± 1.55% (100 nM) (Figure 4). In contrast, 2^′^-modified si-1OMe was not as effective as unmodified si-1, as there was only a slight reduction in EV71 RNA transcripts compared with the mock transfection control (Figure 4). The inhibitory effects of the siRNAs targeting the EV71 genome were shown to be sequence-specific, as reductions in EV71 RNA transcripts were observed to be negligible in RD cells transfected with 100 nM of non-specific scrambled siRNA (Figure 4).

It was of interest to know whether the siRNAs could work after EV71 infection. The RD cells were first infected with 0.01 MOI of EV71 for 1 h, followed by treatment with various siRNAs. Also at 36 h post-infection, viral RNA was extracted from the RD cells. The efficiencies of the various siRNAs in inhibiting EV71 replication were analyzed using the real-time TaqMan RT-PCR assay. Results showed that there were significant reductions in EV71 RNA transcripts in a dosage-dependent manner when the infected RD cells were treated with the unmodified (si-1, si-2) and 2^′^-modified siRNAs (si-1 F, si-2OMe, or si-2 F) after EV71 infection (Additional file 1: Figure S1). Thus, administration of unmodified and appropriate 2^′^-modified siRNAs to RD cells after EV71 infection could still effectively inhibit EV71 inhibition.

### Reduction of EV71-specific proteins by unmodified and appropriate 2^′^-modified siRNAs

**Figure 5:**
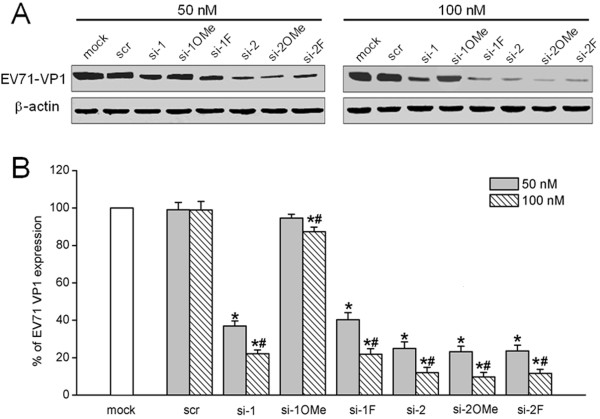
**Western blot analysis of EV71 VP1.** RD cells were transfected with unmodified and 2′-modified siRNAs targeting the 5′ UTR of the EV71 genome and then infected with 0.01 MOI of EV71. At 36 h post-infection, total proteins were extracted from the RD cells. (**A**) Representative western blot of VP1 proteins detected using an anti-EV71 VP1 mouse polyclonal antibody. The internal loading control was detected using an anti-β-actin antibody. (**B**) Quantification of western blot bands was performed using Image J software. The VP1 protein level from the mock transfection control cells was set as 100%. Data were normalized by the β-actin level. All data are presented as means ± SD from three independent experiments. ^*^P < 0.05, compared with mock transfection control. ^#^*P <* 0.05, compared with 50 nM of si-1, si-1OMe, si-1 F, si-2, si-2OMe, and si-2 F, respectively.

In order to investigate whether the reduction in viral RNA transcripts correlated with down-regulation in specific viral proteins, western blotting using a polyclonal antibody against EV71 VP1 structural protein was carried out. EV71 VP1 protein were significantly reduced upon treatment of RD cells with unmodified (si-1, si-2) and 2^′^-modified (si-1 F, si-2OMe, or si-2 F) siRNAs in a dosage-dependent manner (Figure 5). The siRNAs (si-2, si-2OMe, and si-2 F) targeting the 648–666 nt sequence of the EV71 genomic 5^′^ UTR exhibited a significantly greater reduction in the level of detectable VP1 protein compared with siRNAs (si-1 and si-1 F) targeting the 115–133 nt sequence of the EV71 genomic 5^′^ UTR at the same concentrations (Figure 5). The viral protein levels were reduced to 25.00 ± 3.42% and 11.98 ± 2.82% following the transfection of RD cells with 50 nM and 100 nM si-2, respectively, compared with levels in mock transfection control cells (Figure 5B). The 2^′^-modified si-2OMe and si-2 F were also fairly effective in reducing viral protein levels. Viral protein levels evidently declined to 23.28 ± 2.90% and 9.75 ± 2.56% with 50 nM and 100 nM of si-2OMe, respectively. Similarly, viral protein levels declined to 23.64 ± 3.05% and 11.61 ± 2.18% with 50 nM and 100 nM of si-2 F, respectively (Figure 5B). It is noteworthy that transfection with 2^′^-modified si-1OMe resulted in a slight decrease in the synthesis of viral proteins compared with the mock transfection control, obviously different from the unmodified si-1 (Figure 5).

### Reduction of viral titers by unmodified and appropriate 2^′^-modified siRNAs

**Figure 6:**
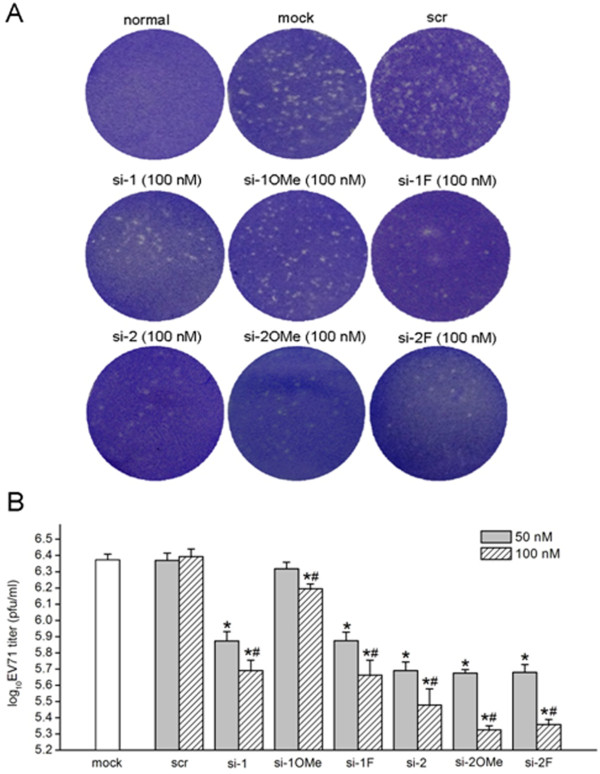
**Determination of virus titers by plaque assays.** RD cells were transfected with unmodified and 2′-modified siRNAs targeting the 5′ UTR of the EV71 genome and then infected with 0.01 MOI of EV71. At 48 h post-infection, culture cell supernatants were collected. Monolayers of RD cells in 6-well plates were inoculated with 500 μl of supernatants at a 10^-4^ dilution for 1 h, and then plaque assays were carried out to determine virus titers. Culture supernatants from non-infected normal cells and mock transfection cells were used as negative and positive control, respectively. (**A**) Representative plaque formation on RD cell monolayers. (**B**) Virus titers in culture supernatants. Values are means ± SD of three independent experiments. ^*^P < 0.05, compared with mock transfection control. ^#^*P* < 0.05, compared with 50 nM of si-1, si-1OMe, si-1 F, si-2, si-2OMe and si-2 F, respectively.

In addition to the examination of the intracellular inhibitory effect of virus RNA replication and VP1 protein expression, the inhibitory effects of the unmodified and 2^′^-modified siRNAs on the yield of EV71 progeny virus were also examined. At 48 h post-infection, virus titers in culture supernatants were quantified by plaque assays. Transfection of RD cells with unmodified (si-1, si-2) and 2^′^-modified (si-1 F, si-2OMe, or si-2 F) siRNAs effectively deceased progeny virus yield, resulting in significant reductions in plaque formation in RD cell monolayers compared with those of the mock transfection control (Figure 6A). Similarly, the siRNAs (si-2, si-2OMe, and si-2 F) targeting the 648–666 nt sequence of EV71 genomic 5^′^ UTR were more effective in reducing viral titers, as evidenced by the reduced plaque count compared with counts observed in the siRNAs (si-1, si-1 F) targeting the 115–133 nt sequence of the EV71 genomic 5^′^ UTR at the same concentrations (Figure 6A). Conversely, RD cells transfected with si-1OMe, even at the concentration of 100 nM, showed only slight inhibition of virus yields with only a limited reduction in the number of plaques (Figure 6A). Transfection with scrambled siRNA did not result in a significant decrease in the viral yield, displaying a similar number of plaques compared with the mock transfection control (Figure 6A). These results reconfirm the sequence-specific inhibitory effects previously observed. RD cells treated with increased concentrations of effective siRNAs were found to have decreased virus yields (Figure 6B) consistent with the results obtained from the real-time TaqMan RT-PCR and western blot analysis.

### Serum stability of siRNAs

**Figure 7:**
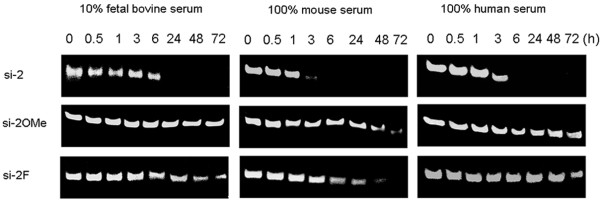
**Serum stability of unmodified si-2 and 2′-modified si-2OMe and si-2 F.** Different siRNAs were incubated in 10% FBS, 100% mouse serum, or 100% human serum at 37°C and withdrawn at the indicated time points. Samples were separated by 15% polyacrylamide-TBE under non-denaturing conditions and visualized by staining with GelRed^TM^ Nucleic Acid Gel Stain. The 2′-modified si-2OMe and si-2 F were still detectable at 48 h of incubation, indicating dramatically enhanced levels of serum stability compared with the unmodified si-2. Three independent experiments were performed.

Unmodified siRNAs are known to be unstable in serum [16]. Therefore, we designed 2^′^-modified siRNAs to determine if these modifications could improve the properties of siRNA for further *in vivo* applications. The serum stabilities of si-2, si-2OMe, and si-2 F were evaluated. When incubated in 10% FBS, undiluted human serum, or undiluted mouse serum, the unmodified si-2 was not detected after 6 h (Figure 7). Conversely, the 2^′^-modified si-2OMe and si-2 F showed striking stability and were detected for at minimum of 48 h under the same conditions (Figure 7). A slight difference in stability was observed between si-2 F and si-2OMe (Figure 7), with si-2OMe showing a significantly greater level of stability in 10% FBS, 100% mouse, and 100% human serum that could be detected after 72 h of incubation (Figure 7). These results clearly indicated that 2^′^-OMe and 2^′^-F modifications could reduce susceptibility to nuclease degradation and dramatically increase the serum stability of siRNAs.

## Discussion

For many years, there has been an ongoing search for effective vaccines or antiviral agents for the treatment of EV71 infections. Previous studies have shown that EV71 for its survival and replication developed a defense mechanism that could not only eliminate the innate interferon immune response but also weaken the effect of exogenous interferon treatment. The 3C protein of EV71 inhibits retinoid acid-inducible gene I-mediated interferon regulatory factor 3 activation and type I interferon responses [33]. EV71 suppresses Toll-like receptor 3-mediated type I interferon responses by down-regulation of Toll/interleukin-1 receptor domain-containing adaptor inducing beta interferon (TRIF) [34]. EV71 also antagonizes type I interferon signaling by reducing the level of interferon receptor 1 [35]. Traditional interferon therapy, which has been successfully applied to many viruses, showed only weak anti-EV71 activities and is not suitable for the treatment of EV71 infections [35,36]. Given the limitations of current therapies for EV71 infections, our attention was drawn to the developing RNAi research field. In this study, unmodified and 2^′^-modified siRNAs were designed to target the highly conserved 5^′^ UTR of the EV71 genome to inhibit EV71 infection *in vitro*. When compared with EV71 China strain genomes available in GenBank in the previous 5 years, siRNAs targeting the 115–133 nt sequence of the EV71 genomic 5^′^ UTR indicated the rates of sequences with complete nucleotide coincidence reached about 71.30%, while the siRNAs targeting the 648–666 nt sequence of the EV71 genomic 5^′^ UTR showed the rates of sequences with complete nucleotide coincidence (37.04%) and those with the same single-nucleotide difference (41.66%) together reached about 79.70% (Additional file 2: Table S1). Thus, siRNA nucleotide sequence analyses indicated that a mixture of the siRNAs (including consideration of the same single-nucleotide mismatch of target sequences to design a pair of siRNAs) targeting these two regions above may have much broader antiviral effect on diverse EV71 China strains.

Transfection of RD cells with siRNAs targeting the 5^′^ UTR of the EV71 genome delayed and alleviated CPE related to EV71 infection and increased cell viability in EV71-infected RD cells. The inhibitory effects against EV71 replication were sequence-specific and dosage-dependent, resulting in significant decreases in viral RNA, VP1 protein and viral titer. The most effective inhibition was observed for the siRNAs (si-2, si-2OMe, and si-2 F) targeting the 648–666 nt sequence of the 5^′^ UTR of the EV71 genome, followed by the siRNAs (si-1 and si-1 F) targeting the 115–133 nt sequence of the 5^′^ UTR of the EV71 genome. It is likely that variations in efficiency are primarily due to differences in positional accessibility of respective target sequences. The current research demonstrates that the highly conserved 5^′^ UTR of the EV71 genome can be targeted for inhibition of EV71 replication by efficient siRNAs. The results were consistent with previous studies of siRNAs targeting the 5^′^ UTR of other RNA viruses such as hepatitis C virus [37,38] and coxsackievirus B3 [20]. These studies demonstrated that siRNAs targeting the 5' UTR of the viral genome can exert significant antiviral activity. However, siRNAs targeting the highly conserved 5^′^ UTR of the poliovirus genome failed to inhibit virus replication [39]. It is possible that some siRNAs cannot efficiently hybridize to target sequences of the viral 5^′^ UTR due to complex secondary structure or protein interactions with RNA motifs that may occlude the target sequences. Thus, the secondary structure of target RNA is an important factor in selecting a suitable target sequence, as it can have a negative effect on RNAi efficiency [40,41].

When compared to previously reported siRNAs and short hairpin RNAs (shRNAs) [27-31], the target sequence located within the highly conserved 5^′^ UTR is a superiority with a reduced tendency to mutate. Nucleotide sequence analyses suggests that these siRNAs may have broader antiviral effects among EV71 China strains than the majority of previously reported siRNAs or shRNAs [27-29,31], because the target selected in majority of previous studies was not conserved in EV71 China strains. Moreover, to tackle the high mutation rate associated with EV71, the siRNAs in our study can be combined with other efficient siRNAs in previous studies simultaneously targeting multiple regions of the genome to increase the efficiency of inhibiting viral replication.

As EV71 infection is transient in nature [27], it is unnecessary to maintain long-lasting viral knockdown effect, which may result in non-specific adverse effects. Synthesized siRNAs used to mediate RNAi against EV71 may be a good strategy for genetic antiviral therapy as it is straightforward and amenable to chemical modification. The 2^′^-OMe and 2^′^-F chemical modifications are classic patterns that can increase binding affinity, nuclease stability and be well-tolerated throughout the duplex [16]. These advantages are important for therapeutic applications. To optimize the properties of siRNAs for further research and clinical applications, 2^′^-OMe or 2^′^-F modification at U and C sequences on siRNA complementary strands were employed in this study. We compared the serum stabilities as well as gene silencing and cytotoxic effects of the unmodified siRNAs and 2^′^-modified siRNAs.

Enhanced serum stability is a major advantage of chemically modified siRNAs that can prolong their activity [16]. Both 2^′^-OMe and 2^′^-F modifications dramatically increased the serum stability of the siRNAs in the present study. Significant enhancements in serum stability of 2^′^-OMe or 2^′^-F modified siRNAs were observed in 10% FBS, 100% human serum, and 100% mouse serum compared with unmodified 21 nt siRNAs. Our research also demonstrated that 2^′^-OMe or 2^′^-F modification could improve siRNA stability and protect against nuclease degradation [42].

Several studies have demonstrated that 2^′^-OMe or 2^′^-F modification at specific positions in the siRNA duplexes were tolerated without loss of RNAi activity [43,44]. In this study, the RNAi activities of 2^′^-modified siRNAs were evaluated. It was found that 2^′^-modified si-1 F, si-2OMe and si-2 F manifested similar RNAi activity in comparison with unmodified counterparts. The cells transfected with si-2OMe and si-2 F targeting the sequence of nt 648–666 within the 5^′^ UTR of the EV71 genome had better RNAi activity than si-1 F targeting the sequence of nt 115–133 within the 5^′^ UTR of the EV71 genome. The results indicated that the RNAi activity of 2^′^-modified siRNAs can be determined based on the primary sequence of the siRNAs. It is worth noting that the 2^′^-modified si-1OMe showed significantly decrease in antivirus activity compared with unmodified counterparts. It is not clear why the patterns of 2^′^-OMe modifications work for one sequence but not for another. Perhaps the introduction of 2^′^-OMe modifications into the si-1 alters the thermostability of the duplex that could interfere with the activity of siRNA. The results also indicated that siRNA modifications are typically sequence dependent, requiring optimization of the modification strategy for each different siRNA sequence when screening for efficient chemically modified siRNAs.

The application of chemically modified siRNAs to therapeutics has raised a number of concerns about their safety. In this study, cytotoxicity assays were used as a global indicator for monitoring adverse effects of unmodified and the 2^′^-modified 21 nt siRNAs. Neither the unmodified nor the 2^′^-modified 21 nt siRNAs showed any significant cytotoxicity in contrast to the mock transfection control up to 72 h post-transfection, even at a high concentration of 100 nM. The results implied that these siRNAs are safe for therapeutic applications.

Our results clearly showed that the 2^′^-OMe and 2^′^-F modifications were substantially compatible with the siRNA machinery. The appropriately 2^′^-modified siRNAs maintained high antiviral activity, were safe and displayed improved bio-stability compared with unmodified siRNAs. The study showed an improvement in triggering RNAi against virus infection using the more potent 2^′^-OMe-modified and 2^′^-F-modified siRNAs. These results also represent an important step in advancing siRNA into a broad range of therapeutic areas.

## Conclusions

EV71 will very likely continue to cause serious problems in hundreds of thousands of young children in the future. In a direct approach using infectious EV71 to challenge siRNA-transfected cells, we demonstrated that the highly conserved 5^′^ UTR of the EV71 genome is an attractive target for inhibition of viral replication by the RNAi strategy. 2^′^-modified siRNA may be more effective and promising than unmodified siRNA as a therapeutic agent against EV71 infection. This study provides new target sequences and highlights the potential of appropriate 2^′^-modifications in improving properties of siRNAs to overcome the barriers to RNAi-based antiviral therapies and to bring RNAi technology to the clinic in the future.

## Abbreviations

(EV71) = Enterovirus 71; (siRNA) = Small interfering RNA; (UTR) = Untranslated region; (RNAi) = RNA interference; (RD) = Rhabdomyosarcoma; (HFMD) = Hand foot, and mouth disease; (2′-OMe) = 2^′^-O-methylation; (2′-F) = 2^′^-fluoro; (nt) = Nucleotide.

## Competing interests

The authors declare that they have no competing interests.

## Authors’ contributions

JXD and XJN participated in the design of the study, performed the experiment, and drafted the initial manuscript. YFL aided in study design and manuscript drafting. CFM aided in siRNA design and participated in the sequence alignment. DLX coordinated the study and performed statistical analyses. BL aided in drafting the manuscript. ZKX and GCZ conceived the study, and participated in its design and coordination throughout. All authors read and approved the final manuscript.

## Supplementary Material

Additional file 1: **Figure S1.** The levels of EV71 RNA transcripts of RD cells transfected with siRNAs after EV71 infection. The RD cells were first infected with 0.01 MOI of EV71 for 1 h, followed by treatment with various siRNAs. At 36 h post-infection, viral RNA was extracted from the RD cells. The EV71 RNA transcript levels are shown relative to the mock transfection control, which was set as 100%. Data are presented as means ± SD from three independent experiments, each performed in duplicate. ^*^P < 0.05, compared with mock transfection control. ^#^*P* < 0.05, compared with 50 nM of si-1, si-1OMe, si-1F, si-2, si-2OMe, and si-2F, respectively. Click here for file

Additional file 2: **Table S1.** Analysis of nucleotide sequence variation corresponding to the siRNA in different EV71 China strains. Click here for file
