# Supplementary material for: The highly conserved 5' untranslated region as an effective target towards the inhibition of Enterovirus 71 replication by unmodified and appropriate 2'-modified siRNAs
Source: J Biomed Sci. 2012 Aug 13;19(1):73. doi: 10.1186/1423-0127-19-73 (PMC3438048; doi:10.1186/1423-0127-19-73)
Supplement: Additional file 1: — Figure S1. The levels of EV71 RNA transcripts of RD cells transfected with siRNAs after EV71 infection. The RD cells were first infected with 0.01 MOI of EV71 for 1 h, followed by treatment with various siRNAs. At 36 h post-infection, viral RNA was extracted from the RD cells. The EV71 RNA transcript levels are shown relative to the mock transfection control, which was set as 100%. Data are presented as means ± SD from three independent experiments, each performed in duplicate. *P < 0.05, compared with mock transfection control. #P < 0.05, compared with 50 nM of si-1, si-1OMe, si-1F, si-2, si-2OMe, and si-2F, respectively. [file 1423-0127-19-73-S1.doc]

**Supplementary Figure 1**

**
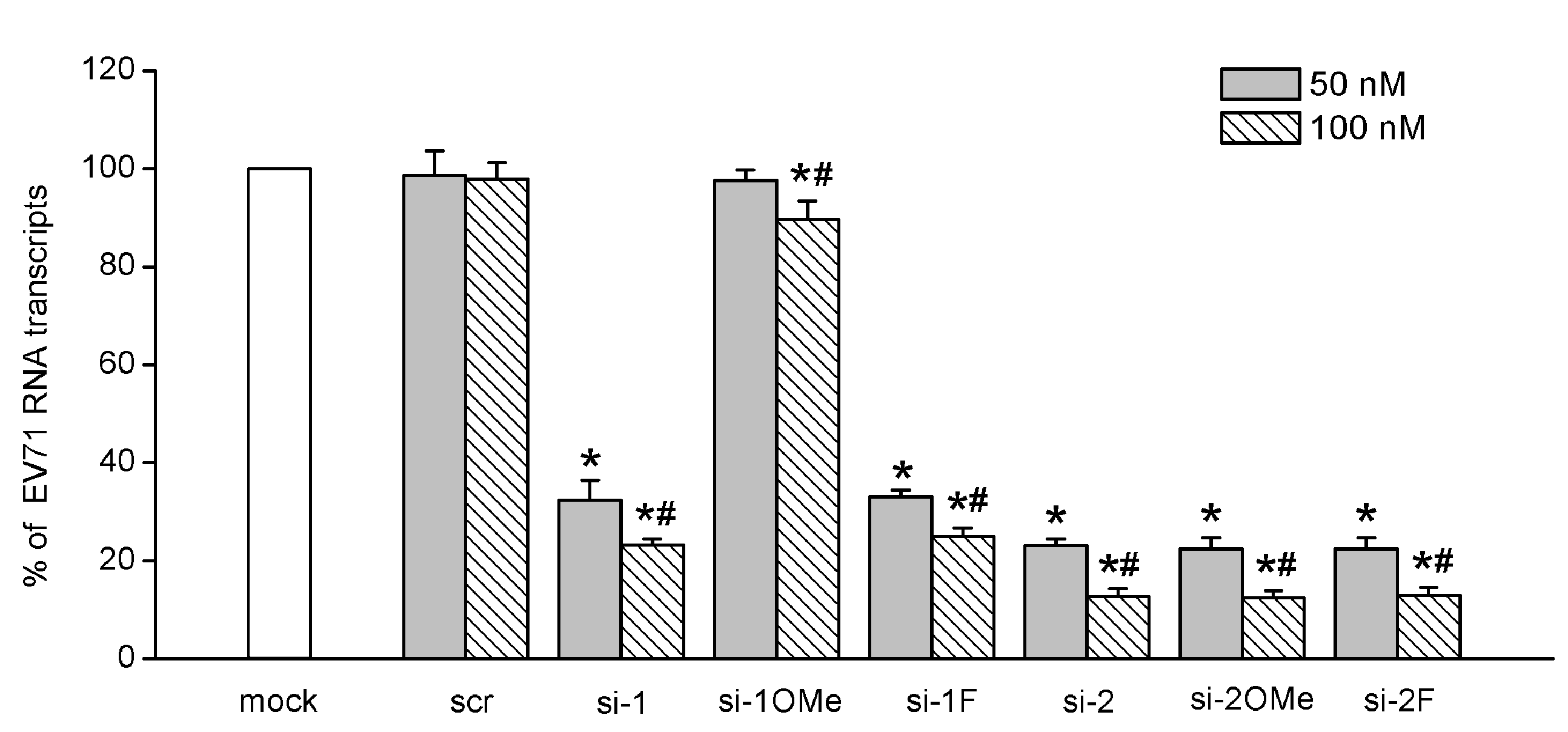
**

**Figure legends**

**Supplementary Figure 1 The levels of EV71 RNA transcripts of RD cells transfected with siRNAs after EV71 infection.** The RD cells were first infected with 0.01 MOI of EV71 for 1 h, followed by treatment with various siRNAs. At 36 h post-infection, viral RNA was extracted from the RD cells. The EV71 RNA transcript levels are shown relative to the mock transfection control, which was set as 100%. Data are presented as means ± SD from three independent experiments, each performed in duplicate. ^＊^*P* < 0.05, compared with mock transfection control. ^#^*P* < 0.05, compared with 50 nM of si-1, si-1OMe, si-1F, si-2, si-2OMe, and si-2F, respectively.
