# Supplementary material for: The highly conserved 5' untranslated region as an effective target towards the inhibition of Enterovirus 71 replication by unmodified and appropriate 2'-modified siRNAs
Source: J Biomed Sci. 2012 Aug 13;19(1):73. doi: 10.1186/1423-0127-19-73 (PMC3438048; doi:10.1186/1423-0127-19-73)
Supplement: Additional file 2: — Table S1. Analysis of nucleotide sequence variation corresponding to the siRNA in different EV71 China strains. [file 1423-0127-19-73-S2.doc]

**Supplementary Table 1 Analysis of nucleotide sequence variation corresponding to the siRNA in different EV71 China strains**

| siRNA targeting the sequence |  | siRNA targeting the sequence |  |
| --- | --- | --- | --- |
| 115 133 |  | 648 666 | Genbank accession |
| CAGCAAACCACGAUCAAUA |  | CAGAGCAAUUGUUUACCUA |
| ------------------- |  | ----------**A**-------- | EU703812.1 |
| ------------------- |  | ----------**A**-------- | EU703813.1 |
| ------------------- |  | ------------------- | EU864507.1 |
| --**A**---**G**------------ |  | ----------**A**-------- | EU812515.1 |
| ------------------- |  | ----------**A**-------- | EU703814.1 |
| -----------------**C**- |  | ------------------- | FJ194965.1 |
| ------------------- |  | ----------------**U**-- | FJ194964.1 |
| ------------------- |  | ------------------- | FJ360546.1 |
| ------------------- |  | ----------**A**-------- | FJ606448.1 |
| --**A**---------------- |  | ------------------- | FJ713137.1 |
| ------------------- |  | ----------**A**-------- | FJ606449.1 |
| ------------------- |  | ----------**A**-------- | FJ606450.1 |
| ------------------- |  | ------------------- | FJ607337.1 |
| ------------------- |  | ------------------- | FJ607335.1 |
| ------------------- |  | ------------------- | FJ607338.1 |
| ------------------- |  | ----------**A**-------- | FJ607336.1 |
| ------------------- |  | ------------------- | FJ607334.1 |
| -------**U**--------**G**-- |  | ------------------**G** | FJ606447.1 |
| ------------------- |  | ----------**A**-------- | FJ439769.1 |
| --**A**---------------- |  | ------------------- | FJ360544.1 |
| ------------------- |  | ------------------- | FJ360545.1 |
| ------------------- |  | ----------**A**-------- | FJ158600.1 |
| ------------------- |  | ----------**A**-------- | FJ158601.1 |
| --**C**---------------- |  | ------------**C**------ | FJ828519.1 |
| ------------------- |  | ------------------- | GQ892830.1 |
| ------------------- |  | ------------------- | GQ994988.1 |
| ------------------- |  | ------------**C**------ | GQ994989.1 |
| **UUUAC**---**ACU**-------- |  | **U**-----**U**---**A**-**A**------ | GQ231942.1 |
| --------**U**---------- |  | ------------------- | GQ231940.1 |
| ------------------- |  | ------------------- | GQ231928.1 |
| ------------------- |  | ------------------- | GQ231938.1 |
| **UUUAC**---**ACU**-------- |  | **U**-----**U**--**CA**-**A**------ | GQ231936.1 |
| **UUUAC**---**ACU**-------- |  | **U**-----**U**---**A**-**A**------ | GQ231934.1 |
| ------------------- |  | ------------------- | GQ231932.1 |
| ------------------- |  | ------------------- | GQ231930.1 |
| ------------------- |  | ------------------- | GQ231926.1 |
| **UUUAC**---**ACU**------**C**- |  | **U**-----**U**---**A**-**A**-----**G** | GQ231943.1 |
| ------------------- |  | ------------------- | GQ231939.1 |
| **UUUAC**---**ACU**-------- |  | **U**-----**U**---**A**-**A**------ | GQ231941.1 |
| ------------------- |  | ------------------- | GQ231937.1 |
| **UUUAC**---**ACU**-------- |  | **U**-----**U**---**A**-**A**-----**G** | GQ231935.1 |
| ------------------- |  | ------------------- | GQ231933.1 |
| ------------------- |  | ------------------- | GQ231931.1 |
| ------------------- |  | ------------------- | GQ231929.1 |
| ------------------- |  | ------------------- | GQ231927.1 |
| **UUUAC**---**ACU**-------- |  | **U**-----**U**---**A**-**A**------ | GQ231925.1 |
| ------------------- |  | ------------------- | GQ279370.1 |
| ------------------- |  | ------------------**G** | GQ279369.1 |
| ------------------- |  | ------------------- | GQ994991.1 |
| -------**U**----**G**------ |  | ------------------- | GQ994992.1 |
| ------------------- |  | ------------------- | GQ994990.1 |
| ------------------- |  | ------------------- | GU366191.1 |
| ---**U**--------------- |  | ----------**A**-------- | GU396280.1 |
| ------------------- |  | ----------**A**-------- | GU198371.1 |
| ------------------- |  | ----------**A**-------- | GU198369.1 |
| ------------------- |  | ----------**A**-------- | GU198367.1 |
| ------------------- |  | ----------**A**-------- | GU198370.1 |
| ------------------- |  | ----------**A**-------- | GU198368.1 |
| **AC**-------- -------- |  | ------**U**-----**A**--**U**--**C** | GU434678.1 |
| --**A**---**G**------------ |  | ----------**A**-------- | GU459071.1 |
| --**A**---**G**------------ |  | ----------**A**-------- | GU459070.1 |
| ------------------- |  | ------------------- | HM003207.1 |
| --**A**---------------- |  | ------------------- | HM002487.1 |
| ------------------- |  | ------------------- | HM002488.1 |
| ------------------- |  | **U**---------**A**-------**G** | HM245928.1 |
| ---**U**-------------**A**-- |  | -----**U**----**A**-------- | HM245927.1 |
| --**A**--**C**------------- |  | ----------**A**-------- | HM053670.1 |
| ---**U**--------------- |  | ----------**A**-------- | HM053669.1 |
| ------------------- |  | ----------**A**-------- | HM002489.1 |
| **U**-**AUG**-------------- |  | ------------------- | HM002485.1 |
| ------------------- |  | ---------**CA**-------- | HM053671.1 |
| --**A**---------------- |  | ------------------- | HM002486.1 |
| ---**U**--------------- |  | ----------**A**-------- | HM002484.1 |
| ------------------- |  | ----------**A**-------- | HQ188292.1 |
| ------------------- |  | ----------**A**-------- | HQ712020.1 |
| ------------------- |  | ----------**A**-------- | HQ882182.1 |
| ------------------- |  | ------------------- | HQ998852.1 |
| ------------------- |  | ----------**A**-------- | HQ825317.1 |
| ---------**U**--------- |  | ----------**A**-------- | HQ828086.1 |
| ------------------- |  | ------------------- | HQ611148.1 |
| ----**G**-------------- |  | ----------**A**-------- | HQ407557.1 |
| --------**U**---------- |  | --**A**---------------- | HQ423142.1 |
| ---------**C**--------- |  | ------------------- | HQ423143.1 |
| ------------------- |  | ------------------- | HQ400942.1 |
| ------------------- |  | ----------**A**-------- | HQ891928.1 |
| ------------------- |  | ---------**CA**-------- | HQ891926.1 |
| ------------------- |  | ----------**A**-------- | HQ891924.1 |
| ------------------- |  | ----------**A**-------- | HQ891929.1 |
| ------------------- |  | ----------**A**-------- | HQ891927.1 |
| ------------------- |  | ---------**CA**-------- | HQ891925.1 |
| ------------------- |  | ----------**A**-----**U**-- | HQ325852.1 |
| ------------------- |  | ----------**A**-------- | HQ426649.1 |
| ------------------- |  | ----------**A**-------- | JF894382.1 |
| ------------------- |  | ----------**A**-------- | JF894383.1 |
| ------------------- |  | ----------**A**-------- | JF894381.1 |
| ------------------- |  | ------------------- | JF799986.1 |
| **U**----------------**C**- |  | ----------**A**-------- | JF913464.1 |
| ------------------- |  | ----**A**-----**A**-------- | JF830007.1 |
| ------------------- |  | ----------**A**-------- | JN001860.1 |
| ------------------- |  | ----------**A**-------- | JN020147.1 |
| ------------------- |  | ------------------- | JN052925.1 |
| ------------------- |  | ----------**A**-------- | JQ514785.1 |
| ------------------- |  | ----------**A**-------- | JQ074190.1 |
| ------------------- |  | ----------**A**-------- | JQ074188.1 |
| ------------------- |  | ----------**A**-**C**------ | JQ074189.1 |
| ------------------- |  | ----------**A**-------- | JQ074187.1 |
| ------------------- |  | ----------**A**-------- | JQ639384.1 |
| ------------------- |  | **U**---------**A**-------- | JQ639383.1 |

*Identical nucleotides are indicated as ellipsis. Different nucleotides are indicated in bold.
